# Trends in Anogenital Wart Diagnoses in Connecticut, 2013-2017

**DOI:** 10.1001/jamanetworkopen.2019.20168

**Published:** 2020-01-31

**Authors:** Anthony E. Yakely, Linda M. Niccolai, Carlos R. Oliveira

**Affiliations:** 1 Department of Epidemiology of Microbial Diseases, Yale School of Public Health, New Haven, Connecticut; 2 Department of Pediatrics, Yale School of Medicine, New Haven, Connecticut

## Abstract

This cross-sectional study examines trends in the diagnosis of anogenital warts over the course of 5 years in Connecticut, a state that achieved moderate uptake of human papillomavirus vaccine.

## Introduction

Anogenital warts (AGWs) are the earliest clinical manifestation of infection with human papillomavirus (HPV). Two vaccines can prevent nearly all cases of AGW.^1^ In 2013, Connecticut reached a significant milestone and became one of the first US states to achieve moderate (>50%) uptake of HPV vaccine in individuals of both sexes.^2^ This study aimed to measure trends in the incident diagnoses of AGW over the course of 5 years after the achievement of moderate HPV vaccine uptake.

## Methods

This study was approved by the institutional review board of Yale University. A waiver for informed consent was granted because this study uses deidentified data. This study follows the Strengthening the Reporting of Observational Studies in Epidemiology (STROBE) reporting guideline for cross-sectional studies.

For this cross-sectional time series, we compiled data electronically from the Yale–New Haven Health System. Data spanned 5 years (January 1, 2013, to December 31, 2017) and were restricted to patients aged 11 to 39 years who had at least 1 health care visit in a diverse set of outpatient clinics (gynecology, oncology, urgent care, dermatology, surgery, and 3 primary care clinics—pediatric, adolescent, and adult). We identified individuals with an AGW-associated visit using several previously described AGW-related codes: (1) a specific discharge diagnosis code for condyloma acuminatum using a combination of the *International Classification of Diseases, Ninth Revision* and *International Classification of Diseases, Tenth Revision* systems, (2) a claim for treatment of viral warts using *Current Procedural Terminology* codes, or (3) a prescription for a medication used to treat AGW using the National Drug Codes.^3,4^ To supplement these code-based case-finding methods, we also reviewed the free-text data fields on the medical records used by clinicians to summarize the reason for the visit. The primary outcome of interest was the proportion of visits with an incident AGW diagnosis. An incident AGW diagnosis was defined as the first occurrence for a given patient of either genital warts being listed as the reason for the visit or as 1 of the listed codes (*International Classification of Diseases, Ninth Revision* and *International Classification of Diseases, Tenth Revision*, *Current Procedural Terminology*, or National Drug Codes).

For the primary analysis, we estimated the overall percentage changes in AGW diagnoses and the mean percentage change with 95% CIs and Cochran-Armitage tests for 6-month intervals. Analyses were further stratified by age, sex, race, insurance status, and vaccine eligibility by birth year. *P* values for comparison of incidence rates (2-sided) and trends (1-sided) were calculated using Poisson regression and Cochran-Armitage tests, with *P* < .05 considered statistically significant for all comparisons. Analyses were conducted using SAS statistical software version 9.4 (SAS Institute) and Joinpoint statistical software desktop version 4.7.0.0 (National Cancer Institute Surveillance Research Program). Data analyses were conducted between August 2018 and July 2019.

## Results

There were 21 713 individuals who had 54 020 visits from 2013 to 2017. The total number of visits in each year ranged from 9725 to 11 678. Across the clinics, the yearly number of visits ranged from 139 to 5009. The sample of patients with a visit during the study period was predominantly female (16 133 patients [74.3%]), had public insurance (15 344 patients [70.7%]), and identified as Hispanic (7587 patients [35.9%]), non-Hispanic black (7589 patients [35.9%]), non-Hispanic white (4054 patients [19.2%]), or non-Hispanic other (1903 patients [9.0%]). A total of 97 incident cases of AGW were identified. The overall proportion of incident AGW diagnoses decreased by 64.5% within the 5-year study period (from 3.1 cases per 1000 visits to 1.1 cases per 1000 visits; incident rate ratio, 0.36; 95% CI, 0.18 to 0.70; *P* = .003). The rate of incident AGW diagnoses was initially higher in male patients than in female patients (5.8 cases per 1000 visits vs 2.3 cases per 1000 visits; incident rate ratio, 2.53; 95% CI, 1.22 to 5.21; *P* = .02), although this difference largely disappeared by the last year of observation (1.8 cases per 1000 visits vs 0.9 cases per 1000 visits; incident rate ratio, 2.03; 95% CI, 0.65 to 6.42; *P* = .23). Decreases in the incidence of AGW were seen among the subgroups of younger (mean percentage change, −40.8% [95% CI, −57.1% to −18.3%] for patients aged 11-19 years and −30.7% [95% CI, −43.4% to −15.1%] for patients aged 20-29 years), vaccine-eligible (mean percentage change, −30.7% [95% CI, −42.8% to −16.1%]), non-Hispanic black (mean percentage change, −32.1% [95% CI, −49.1% to −9.3%]), and non-Hispanic white (mean percentage change, −18.6% [95% CI, −29.9% to −5.6%]) patients (Figure). Similar decreases were observed in both male patients (mean percentage change, −22.2% [95% CI, −35.7% to −5.9%]) and female patients (mean percentage change, −28.3% [95% CI, −42.5% to −10.5%]).

**Figure.**
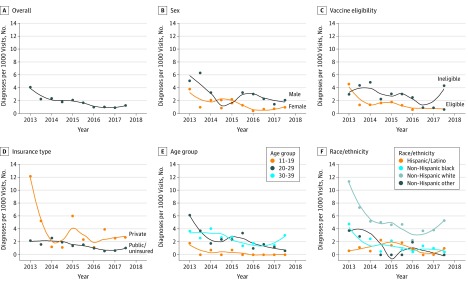
Biannual Trends in Proportion of Visits With Incident Anogenital Warts Graphs show incidence of anogenital warts overall (mean percentage change, −25.1% [95% CI, −32.9% to −16.4%]), by sex (mean percentage change, −28.3% [95% CI, −42.5% to −10.5%] for female patients and −22.2% [95% CI, −35.7% to −5.9%] for male patients), by vaccine eligibility (mean percentage change, −30.7% [95% CI, −42.8% to −16.1%] for eligible patients and −8.7% [−26.2% to 13.0%] for ineligible patients), by insurance type (mean percentage change, −27.9% [95% CI, −45.8% to −4.0%] for private insurance and −22.2% [95% CI, −32.8% to −9.8%] for public insurance or uninsured), by age group (mean percentage change, −40.8% [95% CI, −57.1% to −18.3%] for 11 to 19 years, −30.7% [95% CI, −43.4% to −15.1%] for 20 to 29 years, and −12.8% [95% CI, −26.4% to 3.3%] for 30 to 39 years), and by race/ethnicity (mean percentage change, 2.1% [95% CI, −24.8% to 38.6%] for Hispanic or Latino, −32.1% [95% CI, −49.1% to −9.3%] for non-Hispanic black, −18.6% [95% CI, −29.9% to −5.6%] for non-Hispanic white, and −21.4% [95% CI, −44.8% to 12.1%] for non-Hispanic other). Trend lines were generated by fitting the data with a smoothing function with a local polynomial regression approach.

## Discussion

The United States is among the few countries that have implemented an HPV vaccination strategy that targets both male and female individuals for routine vaccination. Modeling studies have suggested that a gender-neutral immunization strategy could reduce the burden of HPV-related diseases by increasing herd immunity outcomes.^5^ However, real-world evidence that supports the estimations of these models has been limited.^6^ In this study of data from the Yale–New Haven Health System, we found a substantially lower proportion of AGW-related visits in both male and female patients within 5 years of achieving moderate vaccine coverage. The main limitation of this time series is that it could not account for non–vaccine-related factors that could have contributed to changes in diagnoses of AGW (eg, trends in sexual activity, screening practices, or access to care). Furthermore, we only included cases of AGW for which a diagnosis or a treatment was pursued. Thus, these data likely understate the true incidence of AGW in the population. In addition, our data extraction approach may have missed some diagnoses or may have misclassified some cases of AGW. However, one of the strengths in our study was the use of both administrative billing codes and free-text searches, which likely resulted in high and consistent case ascertainment over time.

To our knowledge, these data are the first to demonstrate reductions in AGW that are similar in magnitude in individuals of both sexes. These results may represent protective benefits from a combination of direct outcomes of the vaccine, as well as ongoing herd immunity outcomes associated with the overall increased vaccine uptake. Continual monitoring is needed to determine whether this pattern of coverage can lead to the eradication of AGW.
